# Supplementary material for: Modeling glioblastoma heterogeneity as a dynamic network of cell states
Source: Mol Syst Biol. 2021 Sep 16;17(9):e10105. doi: 10.15252/msb.202010105 (PMC8444284; doi:10.15252/msb.202010105)
Supplement: Supplementary file 6 — Source Data for Figure 5 [file MSB-17-e10105-s004.zip › Figure5A_sourcedata/GSEA_3071/nosigdb_stateIII.GseaPreranked.1622795155562/RICHARDS_NOWAKOWSKI_SCIENCE_2017_MGE_DIV_UPREG.html]

Details for gene set RICHARDS\_NOWAKOWSKI\_SCIENCE\_2017\_MGE\_DIV\_UPREG[GSEA]

|  || Dataset | state33071 |
| Phenotype | NoPhenotypeAvailable |
| Upregulated in class | na\_pos |
| GeneSet | RICHARDS\_NOWAKOWSKI\_SCIENCE\_2017\_MGE\_DIV\_UPREG |
| Enrichment Score (ES) | 0.1728441 |
| Normalized Enrichment Score (NES) | NaN |
| Nominal p-value | NaN |
| FDR q-value | 1.0 |
| FWER p-Value | 0.0 |
Table: GSEA Results Summary

  

Fig 1: Enrichment plot: RICHARDS\_NOWAKOWSKI\_SCIENCE\_2017\_MGE\_DIV\_UPREG      
 Profile of the Running ES Score & Positions of GeneSet Members on the Rank Ordered List

  

| PROBE | GENE SYMBOL | GENE\_TITLE | RANK IN GENE LIST | RANK METRIC SCORE | RUNNING ES | CORE ENRICHMENT || 1 | RPLP0 |  |  | 6 | 0.633 | 0.0060 | Yes |
| 2 | UQCRQ |  |  | 13 | 0.574 | 0.0114 | Yes |
| 3 | RAB13 |  |  | 18 | 0.539 | 0.0168 | Yes |
| 4 | RPL23A |  |  | 27 | 0.502 | 0.0208 | Yes |
| 5 | EEF1B2 |  |  | 28 | 0.501 | 0.0268 | Yes |
| 6 | RPS21 |  |  | 39 | 0.482 | 0.0300 | Yes |
| 7 | RPS6 |  |  | 41 | 0.480 | 0.0355 | Yes |
| 8 | RPL8 |  |  | 44 | 0.469 | 0.0407 | Yes |
| 9 | SNRPC |  |  | 46 | 0.466 | 0.0460 | Yes |
| 10 | NDUFA6 |  |  | 54 | 0.458 | 0.0497 | Yes |
| 11 | CYCS |  |  | 59 | 0.453 | 0.0541 | Yes |
| 12 | COX8A |  |  | 63 | 0.448 | 0.0587 | Yes |
| 13 | RPS3A |  |  | 82 | 0.429 | 0.0591 | Yes |
| 14 | UQCR10 |  |  | 89 | 0.421 | 0.0626 | Yes |
| 15 | RPS27A |  |  | 95 | 0.413 | 0.0662 | Yes |
| 16 | TOMM5 |  |  | 103 | 0.409 | 0.0693 | Yes |
| 17 | CALM2 |  |  | 120 | 0.396 | 0.0698 | Yes |
| 18 | PPIA |  |  | 121 | 0.395 | 0.0746 | Yes |
| 19 | SNRPB |  |  | 122 | 0.395 | 0.0794 | Yes |
| 20 | GNG5 |  |  | 139 | 0.382 | 0.0797 | Yes |
| 21 | RPL21 |  |  | 141 | 0.382 | 0.0840 | Yes |
| 22 | GLO1 |  |  | 161 | 0.367 | 0.0834 | Yes |
| 23 | PSMC3 |  |  | 162 | 0.366 | 0.0878 | Yes |
| 24 | SET |  |  | 163 | 0.366 | 0.0922 | Yes |
| 25 | YBX1 |  |  | 167 | 0.364 | 0.0958 | Yes |
| 26 | PSMB3 |  |  | 171 | 0.361 | 0.0994 | Yes |
| 27 | SNRPG |  |  | 186 | 0.349 | 0.0998 | Yes |
| 28 | PSME1 |  |  | 190 | 0.347 | 0.1032 | Yes |
| 29 | HINT1 |  |  | 205 | 0.335 | 0.1035 | Yes |
| 30 | GSTP1 |  |  | 211 | 0.333 | 0.1062 | Yes |
| 31 | NCL |  |  | 219 | 0.328 | 0.1083 | Yes |
| 32 | RANBP1 |  |  | 221 | 0.328 | 0.1120 | Yes |
| 33 | PTMA |  |  | 222 | 0.327 | 0.1160 | Yes |
| 34 | UQCC2 |  |  | 234 | 0.320 | 0.1169 | Yes |
| 35 | SF3B6 |  |  | 235 | 0.319 | 0.1208 | Yes |
| 36 | ENO1 |  |  | 241 | 0.314 | 0.1232 | Yes |
| 37 | MZT2B |  |  | 244 | 0.313 | 0.1265 | Yes |
| 38 | ACP1 |  |  | 249 | 0.310 | 0.1291 | Yes |
| 39 | SNHG6 |  |  | 266 | 0.303 | 0.1285 | Yes |
| 40 | HNRNPA1 |  |  | 270 | 0.302 | 0.1314 | Yes |
| 41 | TMSB4X |  |  | 273 | 0.300 | 0.1345 | Yes |
| 42 | ANAPC11 |  |  | 286 | 0.295 | 0.1348 | Yes |
| 43 | STMN1 |  |  | 298 | 0.291 | 0.1354 | Yes |
| 44 | TIMM10 |  |  | 304 | 0.290 | 0.1375 | Yes |
| 45 | TPM4 |  |  | 306 | 0.290 | 0.1408 | Yes |
| 46 | PA2G4 |  |  | 308 | 0.289 | 0.1440 | Yes |
| 47 | SRP9 |  |  | 309 | 0.289 | 0.1475 | Yes |
| 48 | NDUFB3 |  |  | 312 | 0.288 | 0.1505 | Yes |
| 49 | HMGB1 |  |  | 318 | 0.286 | 0.1526 | Yes |
| 50 | GAPDH |  |  | 320 | 0.286 | 0.1558 | Yes |
| 51 | SNRPE |  |  | 332 | 0.283 | 0.1563 | Yes |
| 52 | CSNK2B |  |  | 333 | 0.283 | 0.1597 | Yes |
| 53 | UBE2N |  |  | 339 | 0.281 | 0.1618 | Yes |
| 54 | SKA2 |  |  | 343 | 0.280 | 0.1643 | Yes |
| 55 | MYL6 |  |  | 360 | 0.272 | 0.1633 | Yes |
| 56 | CLIC1 |  |  | 373 | 0.269 | 0.1634 | Yes |
| 57 | MRPL51 |  |  | 380 | 0.267 | 0.1650 | Yes |
| 58 | H2AFZ |  |  | 386 | 0.265 | 0.1668 | Yes |
| 59 | RAN |  |  | 388 | 0.264 | 0.1698 | Yes |
| 60 | ZCRB1 |  |  | 395 | 0.261 | 0.1713 | Yes |
| 61 | VPS29 |  |  | 402 | 0.259 | 0.1728 | Yes |
| 62 | HNRNPH1 |  |  | 426 | 0.252 | 0.1697 | No |
| 63 | UQCRH |  |  | 429 | 0.250 | 0.1722 | No |
| 64 | ALDOA |  |  | 440 | -0.251 | 0.1726 | No |
| 65 | MNS1 |  |  | 463 | -0.252 | 0.1697 | No |
| 66 | RAD54L |  |  | 497 | -0.254 | 0.1639 | No |
| 67 | CDC45 |  |  | 506 | -0.255 | 0.1648 | No |
| 68 | SACS |  |  | 510 | -0.255 | 0.1671 | No |
| 69 | HJURP |  |  | 513 | -0.255 | 0.1697 | No |
| 70 | POLE2 |  |  | 520 | -0.255 | 0.1712 | No |
| 71 | LITAF |  |  | 582 | -0.259 | 0.1579 | No |
| 72 | ZNF43 |  |  | 594 | -0.259 | 0.1581 | No |
| 73 | NDC80 |  |  | 636 | -0.261 | 0.1502 | No |
| 74 | MCM4 |  |  | 638 | -0.261 | 0.1531 | No |
| 75 | PRDX2 |  |  | 708 | -0.265 | 0.1378 | No |
| 76 | KITLG |  |  | 723 | -0.266 | 0.1372 | No |
| 77 | JADE1 |  |  | 765 | -0.268 | 0.1295 | No |
| 78 | LINC00342 |  |  | 803 | -0.270 | 0.1228 | No |
| 79 | MSI2 |  |  | 848 | -0.272 | 0.1143 | No |
| 80 | HES6 |  |  | 860 | -0.272 | 0.1146 | No |
| 81 | BCHE |  |  | 867 | -0.272 | 0.1163 | No |
| 82 | HSPA14 |  |  | 896 | -0.274 | 0.1121 | No |
| 83 | ENOSF1 |  |  | 899 | -0.274 | 0.1149 | No |
| 84 | ADCY3 |  |  | 932 | -0.276 | 0.1096 | No |
| 85 | C21orf58 |  |  | 946 | -0.276 | 0.1095 | No |
| 86 | DHFR |  |  | 956 | -0.277 | 0.1104 | No |
| 87 | HELLS |  |  | 970 | -0.278 | 0.1103 | No |
| 88 | MCM3 |  |  | 1021 | -0.280 | 0.1002 | No |
| 89 | CDCA3 |  |  | 1029 | -0.281 | 0.1017 | No |
| 90 | MOB3B |  |  | 1098 | -0.284 | 0.0869 | No |
| 91 | FOXM1 |  |  | 1102 | -0.285 | 0.0896 | No |
| 92 | RNASEH2A |  |  | 1103 | -0.285 | 0.0930 | No |
| 93 | TRIM59 |  |  | 1110 | -0.285 | 0.0948 | No |
| 94 | PCNA |  |  | 1116 | -0.286 | 0.0970 | No |
| 95 | VPS13B |  |  | 1183 | -0.289 | 0.0827 | No |
| 96 | RIF1 |  |  | 1214 | -0.291 | 0.0782 | No |
| 97 | NUSAP1 |  |  | 1262 | -0.293 | 0.0691 | No |
| 98 | DTL |  |  | 1297 | -0.295 | 0.0635 | No |
| 99 | ZNF829 |  |  | 1390 | -0.299 | 0.0424 | No |
| 100 | XRCC2 |  |  | 1435 | -0.302 | 0.0343 | No |
| 101 | GGH |  |  | 1439 | -0.302 | 0.0371 | No |
| 102 | MMS22L |  |  | 1494 | -0.306 | 0.0263 | No |
| 103 | KIF4A |  |  | 1504 | -0.306 | 0.0276 | No |
| 104 | EXO1 |  |  | 1537 | -0.308 | 0.0228 | No |
| 105 | LINC01224 |  |  | 1538 | -0.308 | 0.0265 | No |
| 106 | MYBL2 |  |  | 1587 | -0.311 | 0.0174 | No |
| 107 | PLK4 |  |  | 1642 | -0.315 | 0.0067 | No |
| 108 | ZC3H12C |  |  | 1673 | -0.317 | 0.0024 | No |
| 109 | HAUS8 |  |  | 1741 | -0.321 | -0.0117 | No |
| 110 | NUF2 |  |  | 1755 | -0.322 | -0.0113 | No |
| 111 | WDR76 |  |  | 1795 | -0.324 | -0.0178 | No |
| 112 | BRCA1 |  |  | 1844 | -0.327 | -0.0268 | No |
| 113 | PCDH19 |  |  | 1901 | -0.329 | -0.0378 | No |
| 114 | EFCAB2 |  |  | 1922 | -0.331 | -0.0392 | No |
| 115 | UBE2C |  |  | 1942 | -0.332 | -0.0403 | No |
| 116 | SALL1 |  |  | 1962 | -0.333 | -0.0414 | No |
| 117 | SMC2 |  |  | 1963 | -0.333 | -0.0373 | No |
| 118 | LBR |  |  | 1973 | -0.334 | -0.0357 | No |
| 119 | PKD2 |  |  | 1977 | -0.334 | -0.0325 | No |
| 120 | DNA2 |  |  | 2031 | -0.337 | -0.0426 | No |
| 121 | HUNK |  |  | 2039 | -0.337 | -0.0404 | No |
| 122 | PCDH17 |  |  | 2043 | -0.338 | -0.0371 | No |
| 123 | RRM2 |  |  | 2147 | -0.345 | -0.0606 | No |
| 124 | ZNF704 |  |  | 2210 | -0.350 | -0.0731 | No |
| 125 | LIN9 |  |  | 2212 | -0.350 | -0.0691 | No |
| 126 | BRCA2 |  |  | 2213 | -0.350 | -0.0649 | No |
| 127 | ZNF90 |  |  | 2220 | -0.350 | -0.0622 | No |
| 128 | UHRF1BP1 |  |  | 2238 | -0.351 | -0.0625 | No |
| 129 | ERI2 |  |  | 2254 | -0.353 | -0.0623 | No |
| 130 | FBXW7 |  |  | 2258 | -0.353 | -0.0588 | No |
| 131 | DDIAS |  |  | 2285 | -0.356 | -0.0615 | No |
| 132 | TPX2 |  |  | 2289 | -0.356 | -0.0580 | No |
| 133 | ZNF367 |  |  | 2292 | -0.356 | -0.0542 | No |
| 134 | MCM2 |  |  | 2303 | -0.357 | -0.0526 | No |
| 135 | FRMD5 |  |  | 2309 | -0.358 | -0.0496 | No |
| 136 | ASF1B |  |  | 2312 | -0.358 | -0.0458 | No |
| 137 | MAGI1 |  |  | 2340 | -0.361 | -0.0487 | No |
| 138 | DEPDC1B |  |  | 2354 | -0.362 | -0.0478 | No |
| 139 | SPECC1 |  |  | 2368 | -0.363 | -0.0469 | No |
| 140 | POLQ |  |  | 2401 | -0.365 | -0.0511 | No |
| 141 | IQGAP1 |  |  | 2405 | -0.366 | -0.0475 | No |
| 142 | ZEB2 |  |  | 2484 | -0.372 | -0.0639 | No |
| 143 | BEST3 |  |  | 2518 | -0.375 | -0.0682 | No |
| 144 | ABAT |  |  | 2521 | -0.375 | -0.0642 | No |
| 145 | CDC7 |  |  | 2539 | -0.376 | -0.0642 | No |
| 146 | FAM111B |  |  | 2585 | -0.381 | -0.0717 | No |
| 147 | FADS1 |  |  | 2588 | -0.381 | -0.0677 | No |
| 148 | IQGAP3 |  |  | 2592 | -0.381 | -0.0639 | No |
| 149 | FANCL |  |  | 2644 | -0.385 | -0.0729 | No |
| 150 | KIF20B |  |  | 2689 | -0.390 | -0.0800 | No |
| 151 | CDCA7 |  |  | 2710 | -0.391 | -0.0806 | No |
| 152 | E2F8 |  |  | 2724 | -0.393 | -0.0794 | No |
| 153 | FAM111A |  |  | 2761 | -0.396 | -0.0843 | No |
| 154 | POLH |  |  | 2869 | -0.405 | -0.1081 | No |
| 155 | SMC4 |  |  | 2872 | -0.405 | -0.1037 | No |
| 156 | ZGRF1 |  |  | 2897 | -0.408 | -0.1052 | No |
| 157 | RHOBTB3 |  |  | 2955 | -0.415 | -0.1155 | No |
| 158 | FANCA |  |  | 2976 | -0.417 | -0.1159 | No |
| 159 | CCND2 |  |  | 3037 | -0.425 | -0.1268 | No |
| 160 | ZNF620 |  |  | 3064 | -0.427 | -0.1287 | No |
| 161 | FANCD2 |  |  | 3065 | -0.427 | -0.1235 | No |
| 162 | CCDC150 |  |  | 3069 | -0.428 | -0.1191 | No |
| 163 | RAB3IP |  |  | 3101 | -0.431 | -0.1222 | No |
| 164 | ATAD2 |  |  | 3115 | -0.433 | -0.1205 | No |
| 165 | MCM8 |  |  | 3129 | -0.435 | -0.1187 | No |
| 166 | CCNE2 |  |  | 3138 | -0.436 | -0.1156 | No |
| 167 | PDZRN3 |  |  | 3176 | -0.441 | -0.1202 | No |
| 168 | HSPA5 |  |  | 3193 | -0.442 | -0.1191 | No |
| 169 | NCAPD2 |  |  | 3208 | -0.443 | -0.1175 | No |
| 170 | CDCA2 |  |  | 3212 | -0.444 | -0.1130 | No |
| 171 | SEMA5A |  |  | 3229 | -0.447 | -0.1118 | No |
| 172 | CDCA7L |  |  | 3311 | -0.462 | -0.1280 | No |
| 173 | NRXN3 |  |  | 3318 | -0.464 | -0.1240 | No |
| 174 | POLE |  |  | 3335 | -0.468 | -0.1226 | No |
| 175 | SLC20A1 |  |  | 3344 | -0.470 | -0.1191 | No |
| 176 | BRIP1 |  |  | 3380 | -0.475 | -0.1227 | No |
| 177 | KIF14 |  |  | 3384 | -0.476 | -0.1178 | No |
| 178 | GTSE1 |  |  | 3468 | -0.498 | -0.1340 | No |
| 179 | OMD |  |  | 3481 | -0.501 | -0.1312 | No |
| 180 | NCAPG |  |  | 3513 | -0.512 | -0.1333 | No |
| 181 | DCHS1 |  |  | 3518 | -0.514 | -0.1282 | No |
| 182 | SYNE2 |  |  | 3523 | -0.518 | -0.1230 | No |
| 183 | CDC25C |  |  | 3551 | -0.526 | -0.1239 | No |
| 184 | KIAA1958 |  |  | 3557 | -0.530 | -0.1188 | No |
| 185 | CKB |  |  | 3572 | -0.536 | -0.1161 | No |
| 186 | ILDR2 |  |  | 3592 | -0.544 | -0.1146 | No |
| 187 | SOX2 |  |  | 3613 | -0.555 | -0.1132 | No |
| 188 | CHD7 |  |  | 3622 | -0.561 | -0.1086 | No |
| 189 | ANLN |  |  | 3625 | -0.562 | -0.1023 | No |
| 190 | HIST1H1D |  |  | 3637 | -0.569 | -0.0984 | No |
| 191 | DLX1 |  |  | 3665 | -0.589 | -0.0985 | No |
| 192 | TOP2A |  |  | 3671 | -0.594 | -0.0927 | No |
| 193 | CCNF |  |  | 3690 | -0.608 | -0.0901 | No |
| 194 | CDK6 |  |  | 3700 | -0.614 | -0.0851 | No |
| 195 | CENPE |  |  | 3711 | -0.625 | -0.0803 | No |
| 196 | DLX6 |  |  | 3719 | -0.632 | -0.0745 | No |
| 197 | AKAP12 |  |  | 3740 | -0.660 | -0.0719 | No |
| 198 | DLX2 |  |  | 3742 | -0.662 | -0.0641 | No |
| 199 | FBLN1 |  |  | 3744 | -0.664 | -0.0563 | No |
| 200 | HIST1H1B |  |  | 3749 | -0.669 | -0.0493 | No |
| 201 | RIC3 |  |  | 3785 | -0.718 | -0.0500 | No |
| 202 | MKI67 |  |  | 3788 | -0.721 | -0.0418 | No |
| 203 | CENPF |  |  | 3842 | -0.820 | -0.0462 | No |
| 204 | ASCL1 |  |  | 3852 | -0.843 | -0.0384 | No |
| 205 | PROM1 |  |  | 3854 | -0.846 | -0.0284 | No |
| 206 | ASPM |  |  | 3899 | -1.166 | -0.0261 | No |
| 207 | HIST1H4C |  |  | 3914 | -1.352 | -0.0135 | No |
| 208 | LMO1 |  |  | 3919 | -1.424 | 0.0027 | No |
Table: GSEA details [plain text format]

  

Fig 2: RICHARDS\_NOWAKOWSKI\_SCIENCE\_2017\_MGE\_DIV\_UPREG: Random ES distribution      
 Gene set null distribution of ES for **RICHARDS\_NOWAKOWSKI\_SCIENCE\_2017\_MGE\_DIV\_UPREG**
